# *Tlr4* Deficiency Protects against Cardiac Pressure Overload Induced Hyperinflammation

**DOI:** 10.1371/journal.pone.0142921

**Published:** 2015-11-20

**Authors:** Heidi Ehrentraut, Stefan Felix Ehrentraut, Olaf Boehm, Sakina El Aissati, Fabian Foltz, Lina Goelz, David Goertz, Sied Kebir, Christina Weisheit, Michael Wolf, Rainer Meyer, Georg Baumgarten

**Affiliations:** 1 Department of Anaesthesiology and Intensive Care Medicine, University Hospital Bonn, Sigmund-Freud-Straße 25, 53105, Bonn, Germany; 2 Marienhaus Klinikum, Bad Neuenahr-Ahrweiler, Germany; 3 Asklepios Klinik St. Augustin, Sankt Augustin, Germany; 4 Polyclinic of Orthodontics, University of Bonn, Welschnonnenstraße 17, 53111, Bonn, Germany; 5 Institute of Physiology II, University Hospital Bonn, Nussallee 11, 53115, Bonn, Germany; 6 Klinik für Orthopädie und Unfallchirurgie, HELIOS Medical Center Siegburg, Siegburg, Germany; 7 Department of Neurology, University of Bonn Medical Center, Bonn, Germany; Inserm, FRANCE

## Abstract

Transverse aortic constriction provokes a pro-inflammatory reaction and results in cardiac hypertrophy. Endogenous ligands contribute to cardiac hypertrophy via toll-like receptor (TLR)-4 binding. A lack of TLR4 signaling diminishes hypertrophy and inflammation. Wild type mice undergoing aortic constriction respond to a lipopolysaccharide second-hit stimulus with hyperinflammation. The objective of this study was to assess whether other second-hit challenges utilizing TLR ligands provoke a comparable inflammatory reaction, and to find out whether this response is absent in TLR4 deficient mice. Assuming that cardiac stress alters the expression of pattern recognition receptors we analyzed the effects of transverse aortic constriction and second-hit virulence factor treatment on TLR expression, as well as cytokine regulation. Wild type and *Tlr4* ^*-/-*^ mice were subjected to three days of TAC and subsequently confronted with gram-positive TLR2 ligand lipoteichoic acid (LTA, 15mg/g bodyweight) or synthetic CpG-oligodesoxynucleotide 1668 thioate (20 nmol/kg bodyweight, 30 min after D-galactosamin desensitization) signaling via TLR9. Hemodynamic measurements and organ preservation were performed 6 h after stimulation. Indeed, the study revealed a robust enhancement of LTA induced pattern recognition receptor and cytokine mRNA expression and a LTA-dependent reduction of hemodynamic pressure in TAC wild type mice. Second-Hit treatment with CpG-ODNs led to similar results. However, second-hit effects were abolished in *Tlr4* ^*-/-*^ mice. In total, these data indicate for the first time that cardiac stress increases the inflammatory response towards both, gram-negative and gram-positive, TLR ligands as well as bacterial DNA. The decrease of the inflammatory response upon TLR2 and -9 ligand challenge in TAC *Tlr4* ^*-/-*^ mice demonstrates that a lack of TLR4 signaling does not only prevent left ventricular hypertrophy but also protects the mice from a cardiac stress induced hyperinflammatory reaction.

## Introduction

Severely ill patients demonstrate an increased post-traumatic susceptibility towards secondary bacterial infections [1,2]. Recovering homeostasis after a primary insult is the result of complex mechanisms involving activation and suppression of the immune system. Secondary insults can easily perturb this process. This may cause an inappropriate immune response and increase morbidity and mortality.

Toll-like receptors (TLRs) are a family of pattern recognition receptors (PRR) recognizing pathogen associated molecular patterns such as gram-positive (TLR1, -2, -6) and gram-negative virulence factors (TLR4) as well as bacterial DNA (TLR9). These bacterial TLR ligands may contribute to the pathogenesis of sepsis-induced myocardial inflammation and dysfunction [3–5].

Furthermore, it has been demonstrated that a variety of endogenous ligands signals via TLRs. Those damage associated molecular patterns (DAMPs) such as extracellular matrix components, heat shock proteins, mitochondrial DNA are released upon tissue and cell injury, and activate the immune system via TLRs [6]. In mice, DAMP-induced injury, following cardiac pressure overload and myocardial infarction can be attenuated via inhibition of TLR4 signaling [7–10].

Myocardial injury modulates the cardiac immune system and enhances the subsequent effect of the bacterial virulence factor lipopolysaccharide (LPS) [11,12]. It has been shown that transverse aortic constriction (TAC) alone increases the expression of the TLR4 co-receptor CD14 [12]. However, it remains elusive whether PRRs other than CD14 are also upregulated after TAC. In a second-hit model of pressure overload-induced cardiac hypertrophy followed by endotoxin stimulation, the subsequent LPS challenge induced higher NFκB activation and cytokine expression in the TAC group compared to sham mice, and elevated CD14 expression even further. Polymicrobial sepsis confronts the immune system with a variety of exogenous TLR ligands. In a peritonitis model, the increase of TLR2, 4, and 9 as well as CD14 has been observed [13]. Therefore, we expect that TAC might also alter the response towards ligands signaling via TLR2 and -9. *Tlr4* ^*-/-*^ mice are protected from extensive cardiac hypertrophy. It remains unknown whether TAC influences their cardiac immune response towards TLR ligands such as lipoteichoic acid (LTA) and CpG-ODN 1668 thioate signaling via TLR2 and TLR9.

The purpose of this study was to (i) determine whether TAC changes the sensitivity towards the virulence factors LTA and CpG-ODN, (ii) detect alterations in the expression of pattern recognition receptors after pressure overload-induced hypertrophy and second-hit stimulation; (iii) investigate whether TLR4 deficient mice with reduced susceptibility towards cardiac stress respond with attenuated inflammation towards second-hit LTA or CpG-ODN stimulation.

## Methods

### 2.1 Experimental animals

Experiments were performed on female mice at an age of about 10–12 weeks with an approximate weight of 18-22g. C57BL/6 mice were purchased from Charles River (Sulzfeld, Germany). Breeding pairs of *Tlr4* ^*-/-*^ mice on C57BL/6 genetic background were kindly provided by S. Akira [14]. All animals employed in the present study were housed in individually ventilated pathogen-free cages with free access to water and standard rodent chow. The animals were handled according to the principles of laboratory animal care (NIH publication No. 85–23, revised 1996). The animal procedures carried out in this study were in accordance with German legal guidelines and were specifically approved by the responsible local authority for animal care (Landesamt für Natur, Umwelt und Verbraucherschutz Nordrhein-Westfalen, Recklinghausen, Germany, animal protocols: #50.203.2-BN43 38/06, 84–02.04.2011-A313).

### 2.2 First hit: transverse aortic constriction

Animals were separated into two subgroups, undergoing TAC or sham operation. Aortic banding induced cardiac hypertrophy in mice. Surgery for TAC was achieved as published previously during anesthesia at 2.5 Vol.% isoflurane [11,15]. Mice were intubated in a supine position and mechanical ventilation was initiated (MiniVent 845, Hugo Sachs Elektronik, March-Hugstetten, Germany). Ventilation was adapted to physiological parameters. A left parasternal incision was performed. Retractors were used to achieve a clear sight into the thorax and a suture was passed underneath the aorta and tied down on a 27G needle, which was immediately removed and allowed to achieve a standardized and previously validated decreased diameter of the aorta [11,15]. For sham operation procedure the suture was passed underneath the aorta without ligation. After surgery mice were monitored daily for clinical signs of infection. None of the mice included showed any kinds of healing problems following the surgery. For analgesia mice received a single intraperitoneal (i.p.) injection of 0.065 mg/kg BW buprenorphin.

### 2.3 Second hit: virulence factor treatment

Three days after TAC respectively sham procedure, the impact of cardiac overload on a modulation of the innate immune system was tested. Lipoteichoic acid (LTA; 15mg/kg bodyweight (BW); purified LTA from *S*. *aureus*, endotoxin level <1.25 EU/mg; Cayla-InvivoGen, Toulouse, France) or immunostimulatory CpG-oligodesoxynucleotide (ODN) (1668-thioate; 200 nmol/kg BW = 1.21 mg/kg BW; sequence: GCTAGACGTTAGCGT, TibMolBiol, Berlin, Germany) were i.p. injected. 30min prior to CpG-ODN stimulation, mice were i.p. sensitized with 1mg/kg D-galactosamine (Roth, Karlsruhe, Germany). The CpG-ODN dose was established after mice did not survive stimulation with higher doses (400nmol/kg). Pyrogen-free PBS (Life-Technologies, Gibco BRL, Karlsruhe, Germany) served as control. Previous experiments performed in murine sepsis models did not reveal an impact of D-galactosamine on the inflammatory response [3].

### 2.4 Biometric measurements

The impact on cardiac biometric parameters was investigated 3 d after TAC or sham operation. Body weight was registered. Heart and lung were excised under 2.5 Vol.% isoflurane anesthesia, prepared and total heart weight, left ventricular as well as lung weights and tibia lengths were recorded immediately. Ventricles were snap frozen in liquid nitrogen and kept at -80°C.

### 2.5 Hemodynamic measurements

Hemodynamic parameters were recorded after 3 d of aortic banding followed by 6 h of stimulation using a 1.4 French nylon single pressure micro tip catheter (AD Instruments GmbH, Sperrbach, Germany). Catheter insertion was performed during anesthesia at 2.5 Vol.% isoflurane. Data recordings were performed at 1 Vol.% isoflurane and 1 L/min oxygen flow. For the recording of arterial and left ventricular blood pressure the catheter was inserted into the right carotid artery. First, the catheter was pushed forward to a position 4 mm in front of the aortic valve for peripheral blood pressure recordings and was then further advanced into the left ventricle. Data were analyzed using a power lab data acquisition system (AD Instruments; Software: LabChart for Windows v.6Power Lab).

### 2.6 Quantitative real-time PCR

In order to achieve total RNA extraction, left ventricles were homogenized and RNA was isolated using the thiocyanate-phenol-chloroform method [16]. RNA was dissolved in 100μl of RNase-free water, and concentration was determined photometrically (absorbance at 260 nm) before storage at -80°C. RNA was transcribed reversely according to the manufacturer’s protocol using the High Capacity cDNA Reverse Transcription Kit (Applied Biosystems, Foster City, CA, USA, Part No. 4368814). 25 μl RNA were mixed with 25μl master mix, containing 5 μl 10x reverse transcriptase buffer, 2 μl 25x dNTPs, 2 μl 10x random primers, 2.5 μl multi scribe reverse transcriptase and 10.5 μl nuclease free water.

We used specific pre-made TaqMan^®^ Gene Expression Assays (Applied Biosystems) for GAPDH, ANP, BNP, IL-1β, IL-6, TLR1, -2, -4, -6, -9 (Table 1). Real-time PCR was performed according to the manufacturer’s protocol. 5.5 ng of cDNA was mixed with 5 μl 2xTaqMan^®^ Universal Master Mix (Applied Biosystems, #4304437), 0.5 μl TaqMan^®^ Gene Expression Assay and 2.3 μl nuclease free water to a final volume of 10 μl in a 384-well optical reaction plate. Each sample was measured in triplicate wells and underwent 40 cycles of amplification on an ABI PRISM^®^ Sequence Detection System (Applied Biosystems). C_T_ values were determined with SDS Software 2.2 (Applied Biosystems) and relative quotients (RQ) were calculated following the ΔΔC_T_ method (RQ target gene / GAPDH).

**Table 1:** TaqMan® gene expression assays used for real-time PCR. (TaqMan® Gene Expression Assay, Applied Biosystems).

Primer	Product number
ANP	Mm01255748_g1
BNP	Mm01255770_g1
CD14	Mm00438094_g1
GAPDH	Mm99999915_g1
IL-1β	Mm99999061_mH
IL-6	Mm01210732_g1
IL-10	Mm00439616_m1
IL-1RII	Mm01239300_m1
IL-6Rα	Mm01211445_m1
TLR1	Mm00446095_m1
TLR2	Mm01213946_g1
TLR4	Mm00445273_m1
TLR6	Mm02529782_s1
TLR9	Mm00446193_m1

### 2.7 Data analysis and statistical procedures

All values are expressed as mean (M) ± SEM. For tests of significance between the groups, one-way analysis of variance (ANOVA) and Newman-Keuls *post-hoc* testing were performed. Statistics were calculated using Prism 4.05 (GraphPad Software Inc., San Diego, CA, USA). Differences between experimental groups were considered to be significant with p < 0.05.

## Results

### LTA affects hemodynamic function of TAC operated mice

**Fig 1:**
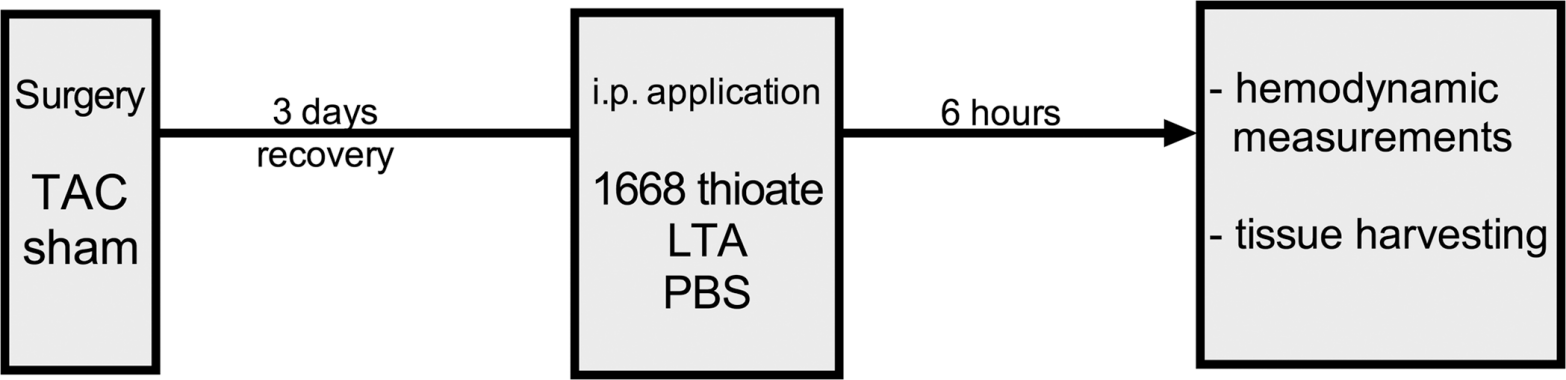
Study design. 3 d after TAC or sham surgery mice were challenged with CpG-ODN 1668 thioate, LTA or PBS as control substance. 6 h after second-hit application, hemodynamic measurements were performed and heart tissue was harvested.

We studied the effect of TAC induced cardiac stress on a subsequent LTA challenge 3 d after surgery in wild type (WT) mice (Fig 1). We confirmed a good reproducibility of TAC surgery. A 17.8% (p<0.01, *n* = 16, PBS groups) and 22.8% (p<0.001, *n* = 21–22, LTA groups) increase of left ventricular weight / tibia length index compared to the respective sham groups was observed which indicates cardiac hypertrophy in both TAC groups.

**Fig 2:**
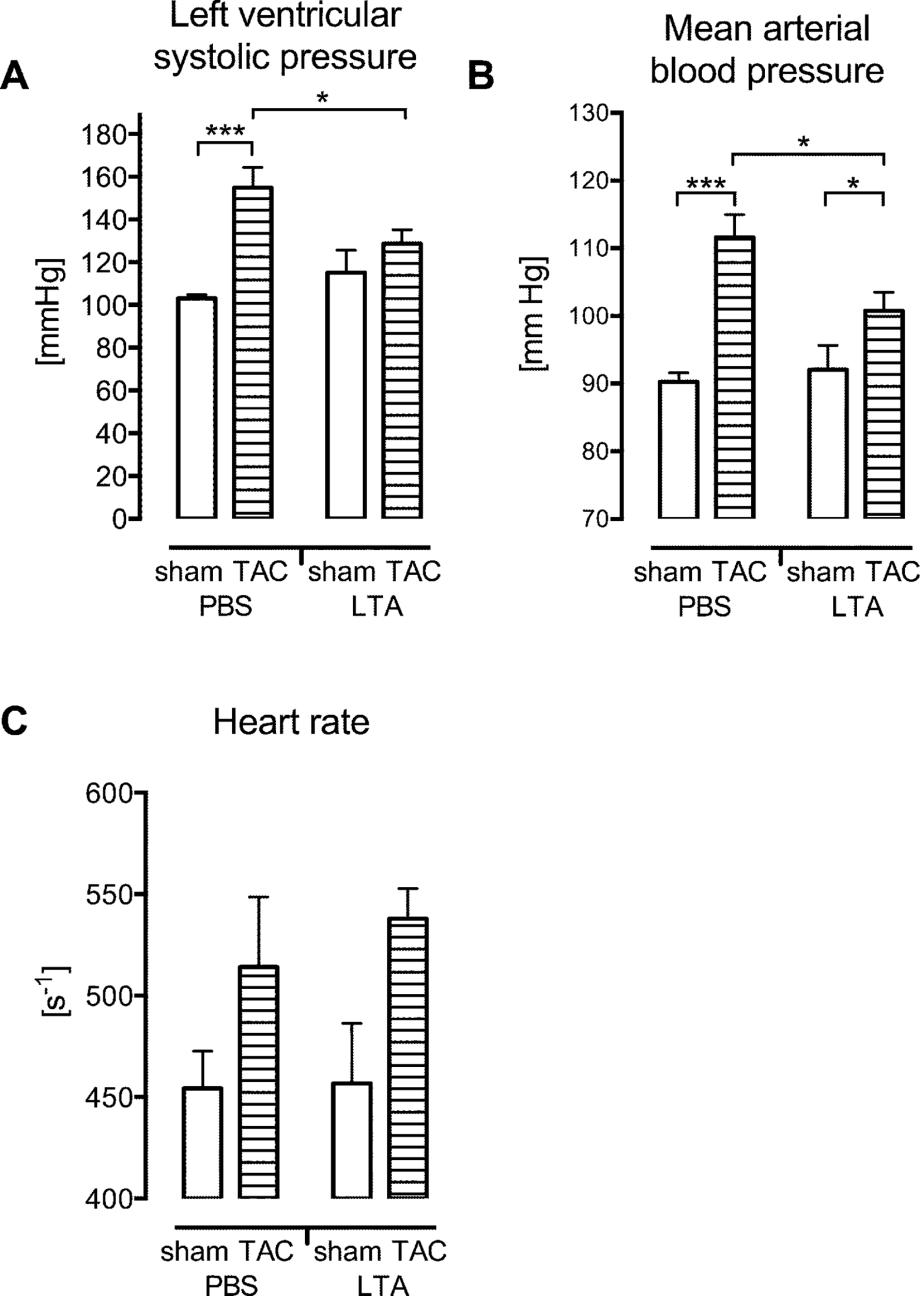
LTA affected the hemodynamic function of TAC operated wild type mice. 3 d of pressure overload increased the left ventricular systolic pressure (A) and mean arterial blood pressure (B) of wild type mice (p<0.001, mean ± SEM, n = 7-13/group). LTA did not cause cardiac depression 6 h after stimulation in sham mice. In TAC pre-treated mice, LTA significantly reduced left ventricular and mean arterial blood pressure (*p<0.05, ***p<0.01). (C) TAC and LTA had no prominent influence on heart rate.

Left ventricular systolic pressure as well as mean arterial blood pressure increased significantly in TAC PBS mice (p < 0.01, Fig 2A and 2B). Six hours after i.p. application, LTA did not cause cardiac depression in the sham group. However, left ventricular systolic and mean arterial blood pressure were reduced in LTA TAC compared to PBS TAC mice (p < 0.05, Fig 2A and 2B) without affecting the heart rate (Fig 2C).

### Cardiac stress changes pattern recognition receptor mRNA expression after LTA treatment

Previously published data demonstrated an elevation of CD14 protein expression after 3 d of TAC, which accounts for increased susceptibility towards an LPS challenge [12]. We hypothesized that myocardial injury and stress could also alter the inflammatory response towards LTA treatment and augment pattern recognition receptor (PRR) expression and signaling.

**Fig 3:**
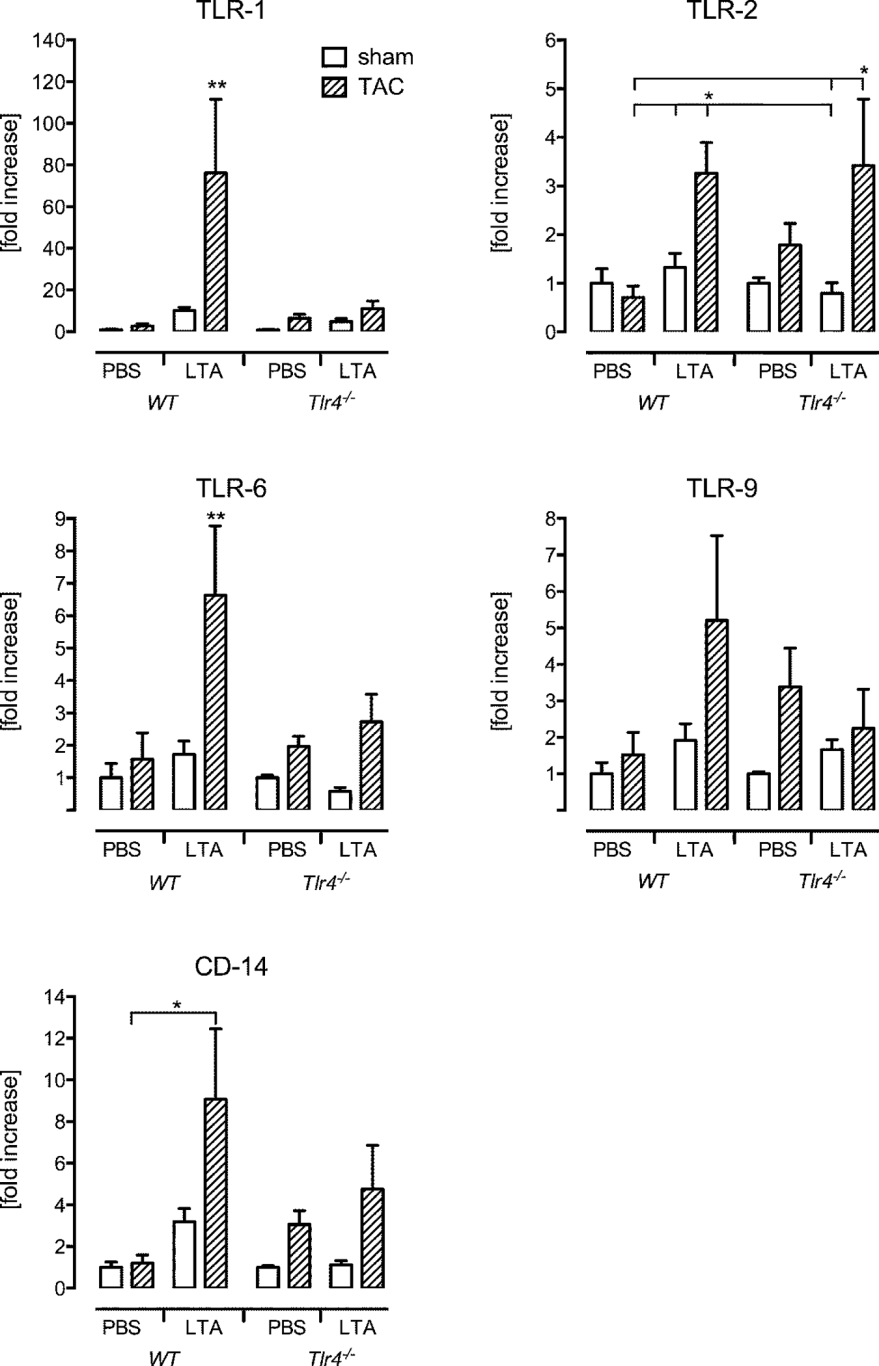
*Differential influence of LTA second-hit treatment on PRR expression in Tlr4* ^*-/-*^ mice. Consecutive LTA stimulation provokes a significant elevation of TLR-1, -2, -6 and CD14 in TAC wild type compared to sham wild type mice. An exclusive upregulation of TLR2 mRNA expression was observed in TAC *Tlr4* ^*-/-*^ mice 6 h after LTA treatment (*p<0.05, **p<0.01, n = 5-6/group).

Indeed, we observed an elevation of TLR-1, -2, -4, -6, -9 and CD14 mRNA amounts in those WT TAC mice confronted with a second hit LTA stimulus, reaching the level of significance except for TLR9 (Table 2, Fig 3). In contrast no major differences between sham PBS and TAC PBS expression were detected on mRNA levels.

**Table 2:** PRR mRNA expression in wild type mice after 3 d of TAC and 6 h of LTA or CpG-ODN 1668 thioate stimulation.

	PBS		LTA		CpG-ODN 1668-thioate	
	sham	TAC	sham	TAC	sham	TAC
TLR1	1.00 ± 0.23*	2.69 ± 1.08*	10.04 ± 1.62*	76.26 ± 35.22*	6.09 ± 2.34	11.12 ± 3.15
TLR2	1.00 ± 0.30*	0.71 ± 0.24*	1.32 ± 0.29*	3.26 ± 0.63*	2.00 ± 1.00	2.90 ± 0.59
TLR4	1.00 ± 0.31*	1.33 ± 0.75	1.71 ± 0.38	3.64 ± 0.90*	2.07 ± 0.52	3.45 ± 0.98
TLR6	1.00 ± 0.44*	1.57 ± 0.82*	1.72 ± 0.41*	6.63 ± 2.15*	1.28 ± 0.50	3.05 ± 0.84
TLR9	1.00 ± 0.30	1.52 ± 0.61	1.92 ± 0.45	5.21 ± 2.32	0.98 ± 0.32	2.52 ± 0.69
CD14	1.00 ± 0.26*	1.20 ± 0.39*	3.19 ± 0.63*	9.06 ± 3.39*	2.45 ± 1.02	4.18 ± 1.26

* p < 0.05 between TAC LTA and indicated groups, n = 5-6/group

### TAC leads to increased cardiac inflammation upon second hit lipoteichoic acid treatment

A previously conducted study elucidated an increased NFκB activation in TAC mice exposed to LPS, followed by enhanced cytokine expression. We assumed that the observed differences in PRR expression presented above might also result in a hyperinflammatory response towards LTA and CpG-ODN.

**Fig 4:**
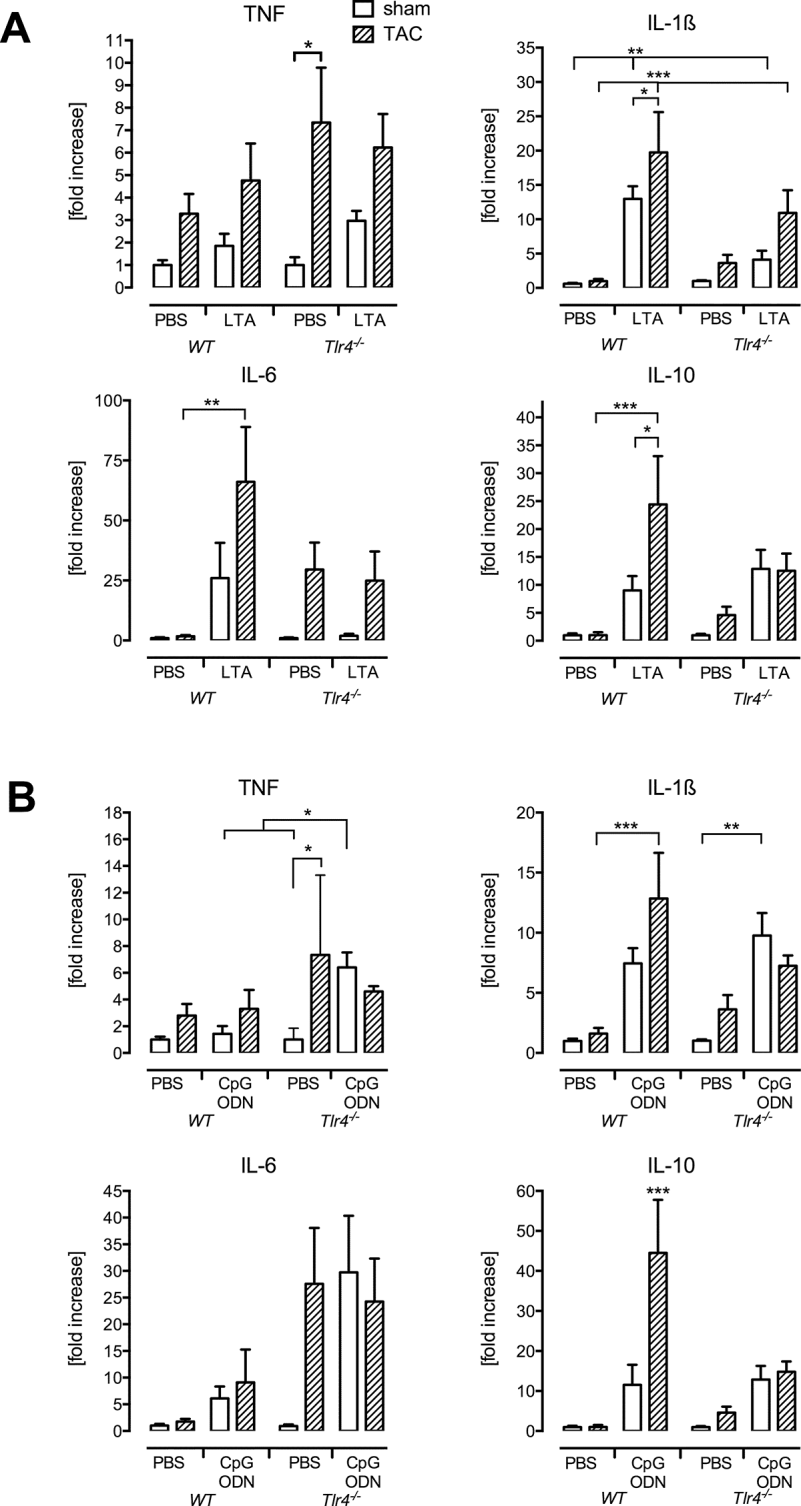
TLR4 deficiency prevents a hyperinflammatory cytokine response upon second-hit virulence factor stimulation. Cytokine mRNA measurements upon LTA (A) or CpG-ODN (B) challenges overall demonstrated a pronounced increase in TAC wild type mice. Cytokine levels upon second-hit treatment did not differ between sham and TAC *Tlr4* ^*-/-*^ mice (*p<0.05, **p<0.01, ***p<0.001, n = 5-6/group).

In line with previously published findings, cytokine expression was not altered anymore 3 d after TAC surgery in wild type mice [11] (Table 3, Fig 4A). Six hours of LTA treatment alone elevated the cytokine mRNA expression, with IL-1β mRNA levels increasing 13-fold compared to PBS sham specimens (p < 0.01). Highest amounts of TNFα, IL-1β, IL-6, and IL-10 mRNA were measured in the TAC LTA second hit group. IL-10 mRNA values significantly exceeded the expression levels in the sham LTA group (p < 0.05), and IL-1β, IL-6 and IL-10 mRNA amounts were significantly elevated compared to TAC PBS (p < 0.001, p < 0.05 and p < 0.01, respectively).

**Table 3:** Cytokine mRNA expression in wild type mice after 3 d of TAC and 6 h of LTA or CpG-ODN 1668 thioate stimulation.

	PBS		LTA		CpG-ODN 1668-thioate	
	sham	TAC	sham	TAC	sham	TAC
TNFα	1.00 ± 0.22	2.80 ± 0.87	1.85 ± 0.54	4.76 ± 1.65	1.43 ± 0.58	3.93 ± 1.56
IL-1β	1.00 ± 0.39 ^a^	1.00 ± 0.30 ^d^ ^e^	12.98± 1.83 ^a^	19.74 ± 5.88 ^d^	4.63 ± 0.79	7.99 ± 2.36 ^e^
IL-6	1.00 ± 0.34	1.75 ± 0.52 ^d^	25.97 ± 14.67	66.09 ± 22.82 ^d^	6.11 ± 2.23	10.76 ± 7.24
IL-10	1.00 ± 0.31	1.00 ± 0.52 ^d^ ^e^	9.02 ± 2.58 ^b^	24.41 ± 8.65 ^b^ ^d^	9.64 ± 4.51 ^c^	44.51 ± 13.26 ^c^ ^e^

Significant differences between relevant groups are indicated by identical superscripted letters (p < 0.05), n = 5-6/group.

a: sham PBS vs. sham LTA

b: sham LTA vs. TAC LTA

c: sham CpG vs. TAC CpG

d: TAC PBS vs. TAC LTA

e: TAC PBS vs. TAC CpG.

### Moderate effect of aortic banding on second-hit CpG induced cardiac inflammation

After observing enhanced inflammation following stimulation with gram-positive and gram-negative bacterial cell wall components, we challenged the immune system with the synthetic bacterial CpG-ODN 1668-thioate signaling via TLR9.

Initially, an amount of 10 nmol CpG-ODN/mouse was utilized but was discontinued due to high mortality. 30% of TAC mice died after stimulation. Out of the surviving mice, 57% died during catheter measurement. Therefore, the concentration was reduced to 5 nmol/mouse with 92% survivors after stimulation and no further loss during catheter procedure.

Expecting a CpG effect on PRR expression we measured the mRNA levels 6 h after 1668-thioate treatment. Highest levels of all PRRs tested were detected in the TAC CpG group. However, the CpG-ODN stimulus was less potent than LTA and the detected mRNA amounts did not reach the level of significance (Table 2).

CpG-ODN caused an increase of cytokines 6 h after stimulation in the sham group. In contrast to previously published data using higher concentrations, significant levels were not detected [3]. Pro-inflammatory cytokine expression data showed an overall elevation but no significant differences between sham and TAC CpG-ODN mice (Table 3). However, IL-10 mRNA levels of TAC CpG-ODN mice exceeded those obtained from the other treatment groups (p < 0.01).

**Fig 5:**
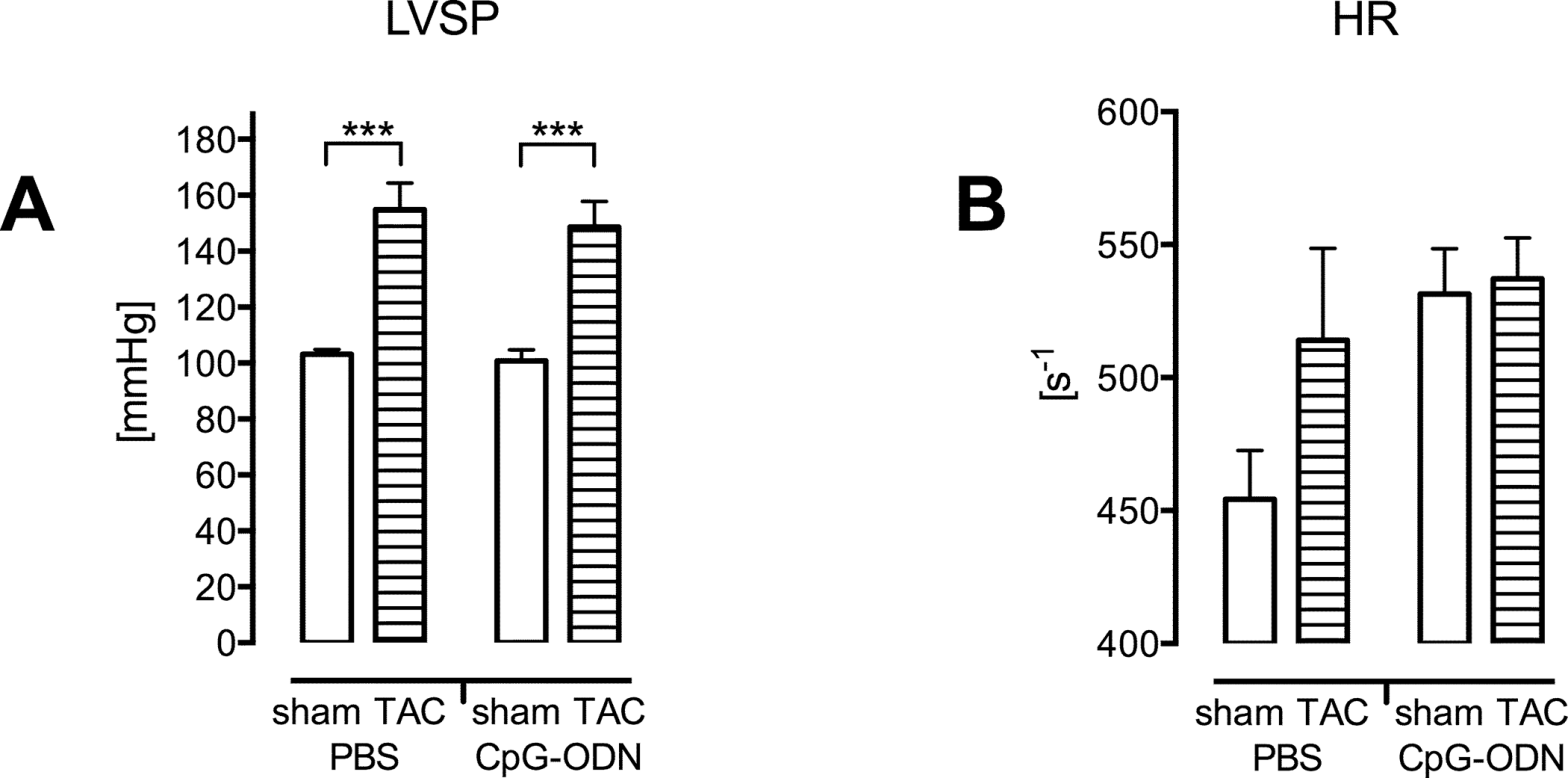
CpG-ODN second-hit challenge of wild type mice has no impact on cardiac function. (A) TAC altered left ventricular systolic pressure (***p<0.001) whereas CpG-ODN stimulation had no influence. (B) Heart rate was only moderately increased after TAC or CpG-ODN challenge (n = 10-14/group).

Five nmol CpG-ODN per mouse had no influence on left ventricular systolic pressure (Fig 5A) whereas high concentrations led to cardiac depression in previous experiments [17]. A moderate increase of heart rate from 454.2 ± 18.32 s^-1^ in PBS to 531.5 ± 17.05 s^-1^ in CpG-ODN sham mice was detected (n.s.; Fig 5B).

### Cardiac function of TAC operated *Tlr4* ^*-/-*^ mice is not altered after LTA or CpG-ODN stimulation

**Fig 6:**
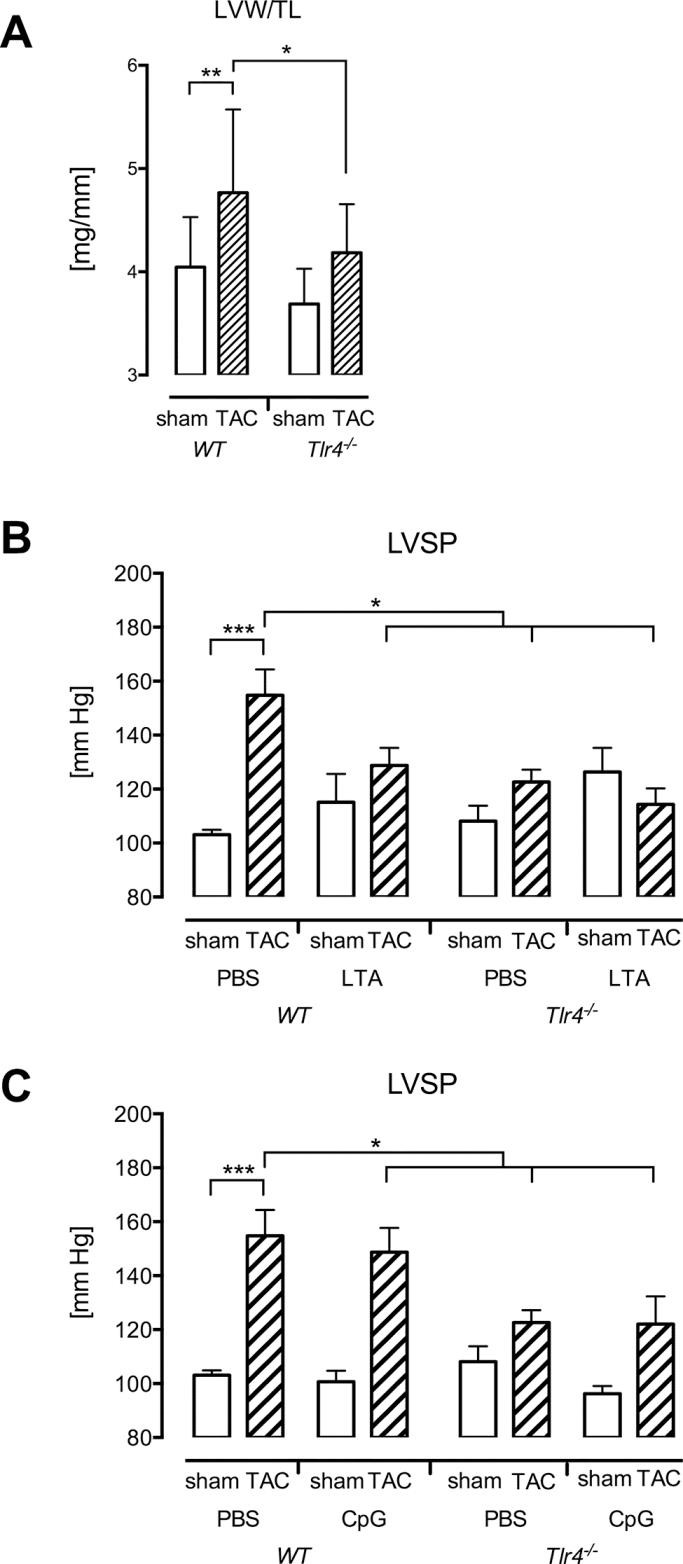
Cardiac function of TAC operated Tlr4^-/-^ mice is not altered after LTA or CpG-ODN stimulation. (A) Reduced left ventricular hypertrophy in female TLR4-deficient mice was confirmed after 3 d of pressure overload (*p<0.05, **p<0.01, ***p<0.001, n = 7–13). In line with this, left ventricular systolic pressure was reduced in PBS TAC *Tlr4* ^*-/-*^ mice (B,C). While LVSP was reduced in TAC LTA wild type mice, LTA or CpG-ODN caused no depression of blood pressure in *Tlr4* ^*-/-*^ mice.

It has been shown that a lack of TLR4 signaling diminishes cardiac hypertrophy following aortic banding [7,8]. We confirmed this finding after 3d of TAC in female mice and observed a significantly reduced left ventricular weight/tibia length index (p < 0.05, Fig 6A) as well as left ventricular systolic pressure (p < 0.05, Fig 6B and 6C) in TAC *Tlr4* ^*-/-*^ mice compared to wild type mice. Second-hit treatment with LTA or CpG-ODNs did not alter left ventricular systolic pressure or other hemodynamic parameters in the TAC *Tlr4* ^*-/-*^ group (Fig 6B and 6C).

### Differential influence on PRR expression after LTA but not CpG-ODN second-hit treatment in *Tlr4* ^*-/-*^ mice

Interestingly, we observed that TAC LTA did not induce significant changes in TLR1, -6, -9 and CD14 mRNA expression compared to the TAC PBS *Tlr4* ^*-/-*^ group (Fig 3). However, TLR2 mRNA expression increased significantly after second-hit LTA treatment compared to *Tlr4* ^*-/-*^ sham LTA (p<0.05). In contrast, second-hit CpG-ODN stimulation did not cause elevated expression of any PRR, including TLR9 (Table 4).

**Table 4:** PRR mRNA expression in *Tlr4* ^*-/-*^ mice after 3 d of TAC and 6 h of CpG-ODN 1668-thioate stimulation.

	PBS		CpG-ODN 1668-thioate	
	sham	TAC	sham	TAC
TLR1	1.00 ± 0.13	6.48 ± 1.87	7.72 ± 2.63	6.40 ± 1.48
TLR2	1.00 ± 0.11	1.78 ± 0.44	5.44 ± 1.52	2.04 ± 0.78
TLR6	1.00 ± 0.08	1.97 ± 0.32	2.57 ± 0.44	1.97 ± 0.29
TLR9	1.00 ± 0.05	3.39 ± 1.06	0.96 ± 0.13	1.69 ± 0.44
CD14	1.00 ± 0.08	3.06 ± 0.66	2.02 ± 0.69	1.64 ± 0.29

n = 5-6/group

### TLR4 deficiency prevents hyperinflammatory state in response to second-hit virulence factor challenge

In *Tlr4* ^*-/-*^ mice second-hit LTA treatment induced only a moderate alteration of cytokine mRNA expression. Detected levels did not significantly exceed the amounts measured in sham LTA or TAC PBS *Tlr4* ^*-/-*^ mice (Fig 4A). Synthetic bacterial DNA also did not cause a hyperinflammatory reaction. CpG-ODN elevated TNF and IL-1β mRNA values in sham mice (p<0.05, Fig 4B). However, mRNA amounts in TAC CpG-ODN mice did not rise above those measured in the sham CpG-ODN group.

## Discussion

We hypothesized that cardiac stress and injury after aortic banding enhance the immune response towards bacterial TLR2 and TLR9 ligands. Furthermore, we assumed that *Tlr4* ^*-/-*^ mice responding to TAC with reduced cardiac hypertrophy and inflammation are protected from a LTA or CpG-ODN induced hyperinflammatory cardiac response. In accordance with these hypotheses our findings indicate that cardiac pressure overload enhances the inflammatory response towards the gram-positive cell wall component LTA and synthetic bacterial DNA. After aortic banding and second-hit LTA stimulation blood pressure decreased while PRR and cytokine expression were elevated. Furthermore, this study demonstrates for the first time that a lack of TLR4 signaling does not only reduce cardiac hypertrophy and inflammation but also protects *Tlr4* ^*-/-*^ mice from hyper-responsiveness towards subsequent virulence factor challenges.

Measurements of hemodynamic and molecular biological parameters were performed 6 h after virulence factor treatment. In a previously conducted investigation applying LPS as a second-hit stimulus, strong alterations of hemodynamic function as well as cytokine secretion were observed 6 h after second-hit application [12]. Due to matters of comparability, we decided to also perform hemodynamic measurements and tissue harvesting 6 h after TLR ligand application. Recent reports revealed that application of ultrapure LTA seems to be a less potent stimulus for cardiac inflammation, depression and vascular contractility compared to LPS [5,13]. NFκB activation and pro-inflammatory cytokine mRNA expression and protein secretion reached their maximum 2–4 h after LTA stimulation and returned to baseline after 6 h. This observation is in line with our results and might explain why only modest alteration of cytokine protein expression was detectable after LTA treatment. Interestingly, in the study presented here application of LTA as a second-hit significantly reduced systolic left ventricular pressure as well as mean arterial pressure. Thus, our investigation revealed that the inflammatory and cardiac depressive effect of LTA is potentiated in a previously injured heart. Therefore, gram-positive bacterial pathogens might be a major contributor to organ dysfunction and inflammation after chronic stress and tissue damage.

Compared to LTA or LPS second-hit stimulation we observed only a moderate inflammatory response and no cardiac depression upon synthetic bacterial DNA challenge. Instead a pronounced increase of anti-inflammatory IL-10 was detected after TAC. In this survey, the effect of CpG-ODN was generally weak. Compared to previous studies, we used a considerably lower dose (200 nmol/kg vs. 400 or 800 nmol/kg bodyweight). Formerly published studies performed with higher CpG-ODN dosages (800 nmol/kg bodyweight) also revealed only a moderate induction of cytokines 6 h after stimulation [3,17], whereas strongest effects were observed after 1-2h post treatment. Cardiac depression was detected in mice injected with 800 nmol CpG-ODN/kg bodyweight after 6h [17]. Therefore, stronger CpG-ODN effects on pro-inflammatory cytokine expression and potential cardiac depression may have been detectable at earlier time points. However, in this study we chose low CpG-ODN concentrations since we observed increased mortality after treatment with 200 nmol CpG-ODN/kg bodyweight.

Former publications demonstrated, that NFκB transcription factor activity and pro-inflammatory cytokine secretion have returned to baseline 3 d past TAC surgery [11,12]. Hence, the hyperinflammatory response is not caused by prolonged increased activity of proinflammatory mediators after the onset of TAC. However, sustained alteration in leukocyte populations occurred during the pathogenesis of LV hypertrophy [18]. CD45^+^ immune cells remained elevated up to 21 d after surgical intervention with monocyte/macrophage and neutrophil numbers increasing significantly on day 3 after TAC. The major proportion of macrophages in the heart was of the Ly6C^low^/M2 type, cells secreting anti-inflammatory cytokines and contributing to tissue repair [19]. In contrast, TAC elevated the amount of Ly6C^high^ monocytes circulating in the blood [18]. These monocytes exhibit phagocytic and pro-inflammatory functions. Thus, an accelerated and increased pro-inflammatory response towards systemically administered bacterial pathogens might arise from a higher abundance of leukocytes in TAC mice. Depending on ligand and concentration, bacterial pathogens induce trained immunity or cause tolerance, partially by epigenetic modification of monocytes [20]. The effects of endogenous TLR ligands remain unknown, but may also alter cellular functional programs and epigenetic events.

We observed that TAC *Tlr4* ^*-/-*^ mice responded with an attenuated inflammatory response towards bacterial pathogen stimulation. TLR4-mediated recognition of host-derived molecules such as fibrinogen activates the immune system and sustained TLR4 activation induces cardiac hypertrophy [21]. Recent studies utilizing different models of cardiac hypertrophy revealed, that MCP-1 and monocyte/macrophage recruitment depended on TLR4 signaling [22,23]. Therefore the lack of leukocyte accumulation in TAC *Tlr4* ^*-/-*^ mice might explain the absence of a hyperinflammatory response upon second-hit treatment in our model.

A recent publication by Jiang *et al*. reported that cardiac signal regulatory protein (SIRP)-α protects against cardiac hypertrophy via the disruption of toll-like receptor 4 signaling [24]. Contrariwise, TLR4-disruption could compensate for the detrimental effects on cardiac hypertrophy in SIRP-α knockdown hearts. SIRP-α is also abundant in immune cells and negatively regulates macrophage activation [25]. These data may suggest a link between TLR4 signal transduction and SIRP-α in our second-hit model. If TLR4-deficient mice exhibit higher SIRP-α levels, this down-regulates innate immune responses. In contrast, TAC wild type mice with reduced SIRP-α levels may develop an augmented, prolonged inflammatory response upon second-hit TLR ligand treatment.

We assumed that pressure overload alters the expression of PRRs. Release of endogenous ligands after TAC with subsequent TLR activation may lead to selective or cross-regulation of TLRs. In TAC wild type mice, LTA TLR2 agonist treatment caused substantial cross-regulation of all PRR mRNA levels measured whereas *Tlr4* ^*-/-*^ mice responded with a differential upregulation of TLR2 mRNA expression. The transcriptional regulation of TLR remains widely unknown. Studies elucidating the negative regulation of TLRs mostly dealt with the dissociation of adaptor complexes, degradation of signal proteins and transcriptional regulation of mediators downstream of TLR [26]. This study now extends this knowledge to the transient regulation of PRRs in a second hit model. Our data here complement the previous findings of PRR regulation in the vasculature during inflammation [13].

Based on our findings, prospective studies will interrogate the regulation of leukocyte recruitment, activation, and function in models of tissue injury predisposing to secondary infections. Mechanistical analyses need to discover whether a transient modulation of TLR4 signal transduction might offer new possibilities for the better use of safe and efficient TLR4 agonists.

## Abbreviations

d: days
DAMP: damage associated molecular pattern
h: hours
IL: interleukin
LPS: lipopolysaccharide
LTA: lipoteichoic acid
ODN: oligodeoxynucleotide
PBS: phosphate buffer solution
PRR: pattern recognition receptor
TAC: transverse aortic constriction
TLR: toll-like receptor
TNF: tumor necrosis factor
